# Stanton Bryan Adams

**Published:** 1947

**Authors:** 


### Obituary

STANTON BRYAN ADAMS, M.B., Ch.B., B.Sc., Ph.D. (Bristol).

STANTON BRYAN ADAMS was born in Bristol in 1904. He was educated at the Bristol Grammar School and the University of Bristol. He entered the Science Faculty in 1922 and graduated B.Sc. with 1st Class Honours in Zoology in 1925. He continued his work in the Department of Zoology and in 1928 was awarded his Ph.D. for a thesis on "The Cervical Nerves of the Platysma in Marsupials." He now realized that his real interest lay in medicine and he entered the Faculty of Medicine in 1928 and qualified M.B., Ch.B. in 1933. During his student career he played a very active part in the work of the Spelaeological Society. In this work he made full use of his training in zoology and earned a considerable reputation in archaeological circles.

After holding various house appointments, he became interested in Radiology and spent a period at the Royal Cancer Hospital, London, studying for the D.M.R. It was here that he became keenly attracted by Radiotherapy to which he subsequently devoted all his interests. Returning to Bristol in 1937 he threw himself body and soul into the work of the recently instituted Radiotherapy Department. Bryan Adams was a real doctor, and to him the best interest of the patient was his only consideration. No detail that might help his patient was too small for him to consider and he never spared himself in his work for them. One of his few complaints during his last illness was the regret he voiced, that he would never be able to let his patients benefit from the experience and knowledge that he gained from his own suffering. Despite the heavy work which the development of the department entailed and the frustration and difficulties occasioned by the war, he still found time to engage in several valuable research problems in radiotherapy.

A loyal and enthusiastic alumnus of his University, he watched its development with great interest. Perhaps as a result of having worked in the Faculties both of Science and Medicine he frequently deplored the tendency of the different faculties to become separate and rather isolated entities. He spent many hours discussing and planning means by which this might be overcome in the bright future which he foresaw for the University and in which he had hoped to play a part.

Bryan Adams had several hobbies. He was an artist in black and white of considerable ability and during the latter years of the war discovered the "scraper board" and became very proficient in this medium. He was a keen and enthusiastic photographer, and obtained much enjoyment in his garden at Maralon Wood. He was a staunch and loyal friend and a good companion in the fullest sense of the term on all and every occasion.

His premature death is a grievous loss to the Royal Hospital, the Region and the University, which all could ill afford. He leaves a widow and two young children to whom we extend our deepest sympathy.

